# Physiological and Behavioral Stress and Anxiety in Children with Autism Spectrum Disorders during Routine Oral Care

**DOI:** 10.1155/2014/694876

**Published:** 2014-07-10

**Authors:** Leah I. Stein, Christianne J. Lane, Marian E. Williams, Michael E. Dawson, José C. Polido, Sharon A. Cermak

**Affiliations:** ^1^Division of Occupational Science and Occupational Therapy, Herman Ostrow School of Dentistry, University of Southern California, 1540 Alcazar Street, CHP 133, Los Angeles, CA 90089, USA; ^2^Division of Biostatistics, Department of Preventive Medicine, University of Southern California, 2001 North Soto Street, SSB 220X, Los Angeles, CA 90089, USA; ^3^Keck School of Medicine of USC, The USC University Center for Excellence in Developmental Disabilities (USC UCEDD), Children's Hospital Los Angeles, 4650 Sunset Boulevard, MS No. 53, Los Angeles, CA 90027, USA; ^4^Department of Psychology, of the Dana and David Dornsife College of Letters, Arts and Sciences, University of Southern California, SGM 3620 McClintock Avenue, SGM 501, Los Angeles, CA 90089-1061, USA; ^5^Children's Hospital Los Angeles, 4650 Sunset Boulevard, MS No. 116, Los Angeles, CA 90027, USA

## Abstract

*Background*. Children with autism spectrum disorders (ASD) commonly exhibit uncooperative behaviors which impede oral care. Previous studies have utilized dentist-report measures of uncooperative behaviors in children with ASD but none have utilized an objective measure of children's behavior or a physiological measure of distress. This study investigated behavioral and physiological distress in children with ASD during routine oral care and examined factors associated with this distress. *Methods*. Participants were 44 children (*n* = 22 typical, *n* = 22 ASD) aged 6–12 receiving routine dental cleanings. Behavioral and physiological measures of stress and anxiety were collected during dental cleanings. *Results*. Children with ASD exhibited greater distress, compared to the typical group, on dentist-report and researcher-coded measures of overt distress behaviors and on physiological measures. Correlations between physiological and behavioral measures of distress were found in the ASD but not in the typical group. Behavioral distress was correlated with age in the typical group and with expressive communication ability and sensory processing difficulties in the ASD group; physiological distress was correlated with parent-report of anxiety in the typical group and sensory processing difficulties in the ASD group. *Conclusions*. Novel strategies may be required to decrease behavioral and physiological distress in children with ASD in the dental clinic.

## 1. Introduction

Children with autism spectrum disorders (ASD) exhibit more dental behavior management problems (uncooperative behaviors) compared to typically developing children, with research indicating that approximately 50–72% of children with ASD exhibit uncooperative behavior during dental treatment [1–3]. These uncooperative and problematic behaviors may include hyperactivity, quick frustration, short attention span, impulsivity, agitation, anger, self-stimulatory, self-injurious, repetitive, aggressive, and disruptive behaviors as well as temper tantrums [4, 5]. Repetitive behaviors and unpredictable, uncontrolled, and impulsive body movements may also complicate dental care by endangering patient safety and posing risk of injury to the dental staff [5].

Autism spectrum disorder is diagnosed based on the presence of specific behavioral criteria including impaired social interaction, social communication, and restricted and repetitive patterns of behavior, interests, or activities [6]. Children with ASD are a heterogeneous group; some are verbally fluent and have average cognitive functioning, while others may have no spoken language and engage in frequent repetitive and self-injurious behaviors [7]. These differences may impact children's ability to cooperate with dental treatment and the degree to which advanced behavior techniques such as restraint or pharmacologic methods may be required [2, 8–10].

Factors contributing to a child with ASD's lack of cooperation may include communication difficulties [3], changes in the child's daily routines [4], sensory sensitivities [11–13], or dental fear and anxiety [14]. Many individuals with ASD also exhibit behavioral difficulties [15] and/or anxiety [16] in addition to the core symptoms of autism, potentially resulting in additional behavior management problems for the dentist.

Uncooperative and aggressive behaviors during dental treatment have the potential to impede, change, or reduce access to care for children with ASD [1–3, 5, 7, 10]. For instance, the greatest barrier to general dentists' willingness to treat children with disabilities is the child's behavior, with 60–80% of dentists stating that they were unwilling to treat patients with developmental disabilities because of their resistive behaviors [17]. Additionally, such behaviors may be the determining factor in deciding if restraint or pharmacologic methods are required and if treatment can occur in the dental office setting or needs to be completed elsewhere (e.g., a hospital under general anesthesia) [2, 8, 18]. In a retrospective study to determine characteristics of patients who were referred for dental treatment in the hospital setting under general anesthesia, behavioral problems had the strongest correlation to dental treatment in the hospital, and the patient having a diagnosis of autism was the fifth strongest characteristic related to location of treatment [18]. Likewise, the use of protective stabilization (i.e., papoose board, restraint by staff or parent, etc.) was significantly associated with uncooperative behavior, after controlling for age and gender in children with ASD [8].

Although overt behavioral displays of distress have been frequently examined, no studies have investigated the physiological stress and anxiety experienced by children with ASD during oral care. Since many children with ASD have limited expressive language skills, it may be especially difficult to assess stress and anxiety due to children's difficulty describing their experience; therefore, utilizing objective tools to measure their physiological experience is an alternative way to obtain this information. Electrodermal activity (EDA) is a non-invasive measure of the ability of the skin to conduct an electrical current, which increases when the sympathetic nervous system is activated [19]. The sympathetic nervous system is the “fight or flight” system, preparing an organism to take action in an emergency or time of stress [20]. It is well-documented that EDA increases in stressful or painful situations [19]; therefore, EDA may be especially useful in measuring responses to the dental experience.

The purpose of this study was to investigate the behavioral and physiological stress and anxiety in children with ASD during routine oral care and determine if there are factors other than ASD diagnosis that are associated with behavioral and physiological distress. Using parent- and dentist-reports of anxiety and uncooperative behavior, video-coding of overt distress behaviors, and physiological recordings of stress and anxiety, we examined three hypotheses: (1) children with a diagnosis of ASD will exhibit significantly greater behavioral and physiological stress and anxiety during routine oral care compared to their typically developing counterparts; (2) physiological stress and anxiety will be correlated with measures of overt distress behaviors; (3) child characteristics (e.g., younger age, lower communication ability, and presence of dental anxiety and sensory processing difficulties) will be significantly correlated with behavioral and physiological stress and anxiety in both ASD and typical children.

## 2. Methods

This study was approved for human participants by the Committee on Clinical Investigations (CCI), the Institutional Review Board for Children's Hospital Los Angeles (CCI-11-00250), and the Institutional Review Board of the University of Southern California Health Sciences (HS-12-00521).

### 2.1. Participants

Forty-four children were recruited for participation in this study, 22 with a diagnosis of ASD and 22 typically developing (TD) as part of the Sensory Adapted Dental Environments to Enhance Oral Health in Children with Autism Spectrum Disorders Study (SADE Study, 1R34DE022263-01). In the SADE Study, ASD and TD groups underwent two dental cleanings in different environments in a counterbalanced manner; only data collected during the cleanings occurring in the regular dental environment are reported here. Eligibility requirements for both the ASD and the typically developing groups were as follows: (1) aged 6 through 12 years, (2) has an accompanying parent/guardian who speaks English or Spanish, (3) is in need of oral cleaning (oral examination and dental prophylaxis, no cleaning within the previous four to six months), (4) does not have a disability such as cleft palate, significant motor impairments (e.g., cerebral palsy), or any known genetic, endocrine, or metabolic dysfunctions that would interfere with oral care or effect EDA, and (5) is willing to participate, able to participate, and has consent of parent/guardian to participate.

Additional inclusion criteria for the ASD group included a confirmed diagnosis of ASD using the Autism Diagnostic Observation Schedule (ADOS), as outlined in the Procedures Section. Additional exclusion for the TD group included (1) diagnosis of ASD or any other developmental disorder, (2) diagnosis of any psychological disorder (e.g., ADHD, clinical anxiety disorder, or bipolar disorder), and (3) sibling diagnosed with ASD. Based on parent report, no children were taking anticholinergic medications which may impact EDA measures [19].

### 2.2. Procedures

Participants were recruited from (1) current and past Children's Hospital Los Angeles (CHLA) dental clinic patients, (2) lists of participants in previous research studies at CHLA who consented to be approached regarding additional research opportunities, and (3) recruitment flyers posted at the CHLA Dental Clinic and local therapy sites for children with ASD.

Parent-report measures to obtain information regarding demographics and children's communication ability, anxiety, and sensory processing were completed in the home prior to the first dental visit (*n* = 26), at CHLA prior to the first dental visit (*n* = 11), or in the home following the first dental visit (*n* = 8) based on parent preference (see details below in Measures section). For children in the ASD group, parents were asked to provide documentation of the professional diagnosis of autism, including a copy of results from an Autism Diagnostic Observation Schedule (ADOS). If the parent did not provide such documentation, children were assessed for this study by a psychologist research-certified in the ADOS to confirm the diagnosis of an autism spectrum disorder.

Approximately 2 weeks prior to the dental cleaning, a social story highlighting the use of “stickers” (electrodermal electrodes) in the dental environment was sent to the participating family; parents were asked to read the story to their children prior to the dental visit in order to increase acceptability of electrode placement on children's fingers. The dental visit consisted of four components: (1) baseline resting period, (2) oral examination, (3) dental prophylaxis (cleaning), and (4) fluoride application.

### 2.3. Measures

#### 2.3.1. Demographics

Demographic information was obtained via the parent-report questionnaire and included child's gender, age, ethnicity, race, and parental education level.

#### 2.3.2. ASD Diagnosis

The Autism Diagnostic Observation Schedule (ADOS) is considered to be the gold-standard for ASD diagnosis [21]. It is a standardized, semi-structured observational measure designed to assess the symptoms important to a diagnosis of autism, including verbal and nonverbal communication, reciprocal social interactions, and repetitive and restricted behaviors and interests. The ADOS shows excellent inter-rater reliability, is internally consistent, and is sensitive and specific in diagnosing ASD.

#### 2.3.3. Communication

Competence in communication was measured by parent-report on the Expressive Language Subtest of the Communication Domain of the* Vineland Adaptive Behavior Scales II *(VABS-II), a norm-referenced, standardized parent-report measure of adaptive functioning [22]. This tool exhibits excellent reliability and validity [22] and has been recommended as a brief measure of expressive language in children with ASD [23].

#### 2.3.4. Anxiety

General anxiety was measured by parent-report on the* Child and Adolescent Symptom Inventory-Anxiety Scale* (CASI-Anx), a 20-item questionnaire assessing a range of anxiety symptoms and scoring on a 4-point Likert scale ranging from 0 (never) to 3 (very often). This measure has been validated on a sample of children with autism aged 5–17 years, including children with IQs below 70 and children without intellectual disabilities [24].

Participant's dental anxiety and past reactions to the dental environment were measured by parent-report on the* Children's Fear Survey Schedule - Dental Subscale *(CFSS-DS) [25]. This tool contains 15 items that pertain to dental treatment; responses range from 1 (not afraid at all) to 5 (very afraid) and are summed for a total score. This assessment has high reliability and validity [26].

#### 2.3.5. Sensory Processing

The* Short Sensory Profile* (SSP) is a standardized screening tool designed to measure children's responses to sensory events in everyday life in multiple modalities (e.g., tactile, olfactory, gustatory, vestibular, auditory, and visual) [27]. This 38-item questionnaire is norm-referenced for children aged from 3 to 10 years. Using 5-point Likert scales, caregivers report how frequently their children respond to sensory input in daily life activities, with lower scores representing greater difficulty in sensory processing. The Sensory Profile has high reliability and validity, is available in English and Spanish, and is one of the most frequently used assessments of sensory processing in children with ASD.

#### 2.3.6. Overt Anxiety and Distress Behaviors

The* Anxiety and Cooperation Scale* (A & C Scale) has been shown to assess children's anxiety, fear, and cooperation as rated by dentists and has good established reliability and validity [28, 29]. Following a routine dental cleaning, the dentist rated overall patient behavior during treatment using a one-item Likert scale ranging from 0 (is relaxed, is smiling, demonstrates desired behavior, and complies with demands) to 5 (out of control, loud crying, reverting to primitive flight responses, and physical restraint required).

The* Frankl Scale* was completed by the dentist following the dental cleaning [30]. This one-item Likert Scale ranges from 1 (definitely negative) to 2 (negative) to 3 (positive) to 4 (definitely positive). This assessment has high inter-rater reliability and moderate validity [31] and has been used to measure the behavior of children with ASD [3, 8].

The* Children's Dental Behavioral Rating Scale* (CDBRS) is a tool developed for the larger NIDCR-funded study in order to evaluate overt distress behaviors exhibited by children during dental care. The child's behavior was videotaped during the dental cleaning; the first five minutes of prophylaxis were coded from the video data at a later date. Coding included marking the presence or absence of three distress behaviors (mouth movement, head movement, and forehead movement) and the presence or absence and severity of two distress behaviors (whimper/cry/scream and verbal stall or delay) during each one-minute interval of the five-minute video. Inter-rater reliability by two trained raters on a sample of 15 children with and without ASD (35% of total sample) was *K* = .97, *P* < .001. The raw score (0–45) was converted, via Rasch analysis, to a scale score of 1–100.

The* number of hands* required to restrain the child during the dental cleaning experience was also utilized as a measure of uncooperative behavior. This variable was recorded on researcher notes during the dental cleaning and was verified using the videotape of the dental cleaning. Scoring included presence/absence as well as the number of hands used for restraint purposes during the cleaning. For these analyses, the number of hands used to restrain children was dichotomized (absent/present) due to the small number of incidences of hands being used in the TD group.

#### 2.3.7. Physiological Stress and Anxiety

Physiological stress and anxiety were measured using electrodermal activity (EDA). EDA was measured during a three-minute rest period prior to the dental cleaning (baseline) as well as throughout the entire dental cleaning (oral exam, prophylaxis, and fluoride application). Two silver-silver chloride pre-gelled disposable electrodes were placed on the distal phalynx of digits two and three of the child's non-dominant hand. EDA was then recorded by connecting the electrodes to the BIOPAC MP150 system. In longer-lasting situations, such as a dental cleaning, measurement of tonic skin conductance level (SCL) and frequency of non-specific skin conductance responses (NS-SCRs) are the most useful electrodermal measures [19]. EDA components, such as SCL and NS-SCRs, exhibit significant test-retest reliability (temporal stability) for control and ASD populations (*r* ranges from .40 to .85) when measured over a duration of a few weeks to a year or longer [19, 32]. Additionally, measurements of EDA have been utilized to investigate clinical populations' responses to stimuli, including responses in participants with ASD [32–34].

### 2.4. Data Analysis

The research design consisted of a comparison of the behavioral and physiological indices of stress and anxiety between children with ASD and TD children during a routine dental cleaning. Correlations between physiological and behavioral measures were examined as well as correlations between these measures and other child characteristics such as age, expressive communication level, general and dental anxiety, and sensory processing.

Electrodermal data collected during both the baseline and dental cleaning periods were analyzed. As is common practice, tonic SCL was transformed prior to analysis to reduce the skew and kurtosis of the data with a logarithmic transformation [19]. The number of non-specific skin conductance responses (NS-SCRs) was totaled for each participant and converted to the rate of fluctuations per minute; NS-SCRs were counted conservatively, only when the amplitude was greater than or equal to .05 *μ*S, as suggested by Dawson et al. [19]. Both SCL and NS-SCRs were computer scored offline using the BIOPAC program Acq*Knowledge*, an interactive program which allows measurement and transformation of EDA data. However, as is standard in EDA analyses, recordings were checked by hand to ensure no skin conductance responses were missed or incorrectly marked; 25% of the hand-coded data were double coded to ensure that the identification of NS-SCRs was reliable, with 96% agreement (calculated as the number of matching NS-SCRs divided by total number of NS-SCRs coded by the researchers).

Data were analyzed using the SPSS computing package (V.21). Participant characteristics for each group were described as Mean (SD) for continuous outcomes and *N* (%) for categorical outcomes. Comparison between groups (TD versus ASD) in stress and behavior variables was performed using ANCOVA models for continuous variables and logistic regression for dichotomous outcomes:* a priori* covariates included visit number (1 or 2), which was randomized in the SADE Study. As this was a pilot study with a small sample size, analyses that approached significance at* P* ≤ .10 were considered meaningful. In addition to significance level of statistics, effect sizes (Cohen's d for mean differences and odds ratio for dichotomous outcomes) adjusted for visit order were computed to help inform the clinical significance of the results. Spearman non-parametric correlation coefficients were calculated to test the relationship between EDA variables (SCL and NS-SCR frequency), measures of overt uncooperative behaviors, and child descriptor variables (age, anxiety, communication ability, and sensory processing difficulty).

## 3. Results

There were no significant differences between the ASD and TD groups in age, ethnicity, race, and maternal and paternal education status. Gender distribution was significantly different between groups (*P* = .01) but consistent with national statistics [35], with the ASD group having more males than females (*n* = 18 : 4) and with a male to female ratio of 4.5 : 1; in the typical group, gender was balanced (*n* = 10 male, *n* = 12 female). See Table 1.

Child-descriptor variables were significantly different between ASD and TD groups. As expected, expressive language ability was significantly lower in children with ASD compared to TD children (*P* < .001). Children with ASD were also reported by their parents to have significantly more difficulties with sensory processing (SSP), general anxiety (CASI-Anx), and dental anxiety (CFSS-DS) compared to TD children (*P* < .001 for all variables). See Table 1.

### 3.1. Between Group Differences in Distress

#### 3.1.1. Overt Behavioral Distress

ASD diagnosis exerted a strong effect on overt behavioral distress exhibited during dental cleaning. As predicted, children with ASD exhibited greater uncooperative behavior during dental care, compared to the TD group, based on dentist-report on the Anxiety and Cooperation Scale and the Frankl Scale (both* P*'s < .001), as well as overt behaviors coded by the CDBRS (*P* = .001). Lastly, the ASD group had a higher incidence of requiring restraint during the dental cleaning compared to TD children (*P* < .001), with only 9% (*n* = 2) of TD children requiring one or more restraining hands versus 73% (*n* = 16) of children with ASD. See Table 2.

#### 3.1.2. Physiological Stress and Anxiety

ASD diagnosis exerted a strong, significant effect on non-specific skin conductance response (NS-SCR) frequency but only a moderate, nonsignificant effect on skin conductance level (SCL). Children with ASD exhibited significantly more frequent NS-SCRs (*P* = .001) compared to TD children during their entire dental cleaning, but there was no significant overall difference between groups when comparing SCL. See Table 2.

**Figure 1:**
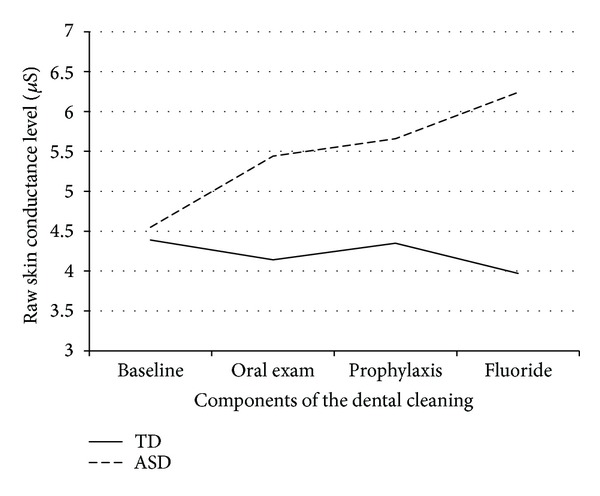
Skin conductance level throughout different components of the routine dental cleaning.

When examining each phase of the dental cleaning separately, children with ASD exhibited significantly greater NS-SCR frequency compared to TD children during the oral exam and prophylaxis as well as during fluoride application (*P*'s = .001, .02, and .05, resp.). As for the SCL measure, children with ASD exhibited an increase in average skin conductance level throughout the dental cleaning process, which was not seen in the TD group (See Figure 1). Additionally, SCL was significantly different between the two groups during fluoride application, with children with ASD exhibiting significantly higher SCL than their TD counterparts, consistent with greater stress and anxiety in the ASD group during this phase of the dental cleaning (*P* = .03).

### 3.2. Correlations between Behavioral and Physiological Measures of Distress

In the TD group (see lower left half of Table 3) the majority of overt behavioral distress measures, both dentist-reported and researcher-coded, were not significantly correlated with EDA variables (SCL and NS-SCR frequency). The exception was SCL during dental cleaning and the dentist-report Anxiety and Cooperation Scale (*r* = −0.37, *P* = .09); however, this correlation was in the opposite direction than expected, indicating that the more physiological stress experienced (the higher the EDA), the less overt behavioral distress exhibited.

In the ASD group (see upper right half of Table 3), the frequency of NS-SCRs was significantly correlated with the dentist-report Anxiety and Cooperation Scale (*r* = 0.62, *P* = .002) and Frankl Scale (*r* = −0.57, *P* = .006), the researcher-coded CDBRS (*r* = 0.43, *P* ≤ .05), and the number of hands required to restrain the child during treatment (*r* = 0.37, *P* = .09). These correlations suggest that as children with ASD were more physiologically stressed (NS-SCRs increased), their overt behavioral distress also increased (higher score on Anxiety and Cooperation Scale, CDBRS, number of hands, and lower score on Frankl Scale). In contrast, SCL was not correlated with any behavioral indices of distress.

### 3.3. Correlations between Child Characteristics and Distress Variables

In the TD group (see lower left half of Table 3), the only child characteristic that was significantly correlated with overt behavioral distress was age; the younger the typical child's age, the greater the uncooperative behavior exhibited on dentist-report measures (A & C Scale: *r* = −.38, *P* = .08; Frankl Scale: *r* = .42, *P* = .05). In the TD group, no relationships were found between parent-reports of the child's general anxiety, dental anxiety, or expressive communication ability and overt behavioral distress as rated by the dentist or video-coding. Although the short sensory profile total score was not significantly correlated with behavioral distress, the tactile sensitivity and movement sensitivity subtests were significantly correlated with the CDBRS (*r* = −.44,  *P* < .04, *r* = −.39, *P* < .08, resp.). Physiologically, only parent-reports of dental anxiety and general anxiety were significantly correlated with physiological distress in TD children (CFSS-DS and SCL: *r* = .38, *P* = .08; CASI-Anx and NS-SCR: *r* = .36, *P* ≤ .10).

In the ASD group (see upper right half of Table 3), expressive communication was significantly correlated with three measures of overt distress behavior (A & C Scale: *r* = −.42, *P* ≤ .05; Frankl Scale: *r* = .44, *P* = .04; restraining hands: *r* = −.68, *P* = .001); the lower the communication ability, the greater the uncooperative behavior observed and reported. Additionally, the correlation between distress behavior on the CDBRS and sensory processing total score also approached significance (*r* = −.35, *P* = .11), with the greater the parent-reported sensory processing difficulty (lower score), the greater the exhibited distress behavior. When investigating the sensory processing subtests, the difficulty with auditory filtering subtest and visual/auditory sensitivity subtest were significantly correlated with behavioral distress [(*Auditory Filtering and:* A & C Scale: *r* = −.46 (*P* = .03); Frankl Scale: *r* = .40 (*P* < .07); CDBRS: *r* = −.44 (*P* = .04);* Visual/Auditory Sensitivity and:* A & C Scale: *r* = −.51 (*P* < .02); Frankl Scale: *r* = .41 (*P* < .06); CDBRS: *r* = −.44 (*P* = .04); restraining hands: *r* = −.37 (*P* < .10)]. In regard to physiological distress in the ASD group, no parent-report measures of general anxiety, dental anxiety, or expressive communication level were significantly correlated with physiological distress during the dental cleaning. Although sensory processing difficulty total score was not correlated with physiological distress, the visual/auditory subtest was significantly correlated with both SCL (*r* = −.51, *P* = .02) and NS-SCR frequency (*r* = −.66, *P* = .001). A correlation approaching significance was found between age and NS-SCR frequency (*r* = −0.35, *P* = .11), indicating that the younger the child with ASD, the greater the physiological distress experienced during his/her dental cleaning.

## 4. Discussion

As hypothesized, children with ASD, compared to TD children, exhibited significantly greater behavioral distress during routine oral care. This finding is consistent with previous research investigating the uncooperative behaviors exhibited by children with ASD during dental care [2, 3, 8]. It is important to note that both the Anxiety and Cooperation Scale and Frankl Scale are Likert-Scale measures with broadly defined items subject to dentist bias; therefore, as no prior studies of children with ASD have utilized an objective, psychometrically sound behavior-coding scale, this study also provides validation for previous research that used only dentist reports of children's uncooperative behavior. When conducting research studies to examine behavior changes, objective behavior coding scales such as the CDBRS may be a useful additional outcome measure.

The hypothesis that children with ASD would exhibit greater physiological distress compared to TD children was endorsed for NS-SCR frequency but not for SCL. This partially supports our assertion that children with ASD would find dental cleanings more aversive than TD children, activating their sympathetic “fight or flight” nervous system in this time of stress [19]. Interestingly, the only other study that has investigated EDA responses to dental cleanings found the opposite; children with developmental disabilities (not ASD) were physiologically less aroused compared to TD children during dental cleanings [36]. However, that study did not include children with ASD and did not utilize standard EDA techniques for measurement or analysis, making it difficult to directly compare to our results.

Also noteworthy is that NS-SCR frequency during dental cleaning shows larger and more consistent correlations with the variables investigated in this study as compared to SCL. Although SCL and NS-SCR frequency are generally accepted as being highly correlated [19] and were found to be highly correlated in this study as well, relationships between NS-SCR frequency and other variables were found when there were none between SCL and variables. This decoupling of SCL and NS-SCR frequency has been reported before. For example, Dawson et al. [37] found heightened SCL in patients with schizophrenia prior to a psychotic relapse, compared to patients with schizophrenia with continued symptomatic remission; NS-SCR frequency did not discriminate between relapse and continued remission patients.

In children with ASD, relationships were found between physiological and behavioral manifestations of distress; children who are more physiologically stressed likewise exhibit greater uncooperative distress behaviors. However, this relationship was not found in the TD group; children who were more physiologically distressed did not exhibit more uncooperative behaviors. This finding may suggest that typically developing children experience physiological stress but use coping strategies, such as cognitive self-talk skills, to calm themselves. For instance, in a study by van Meurs et al. [38], researchers found that typical children in both high caries and low caries prevalence areas believed that thoughts such as “I tell myself I have to do this because it is good for my teeth” and “I tell myself it will be over soon” were very effective coping strategies during dental care. Although we did not ask children to complete the Dental Cope Questionnaire [39] or ask what they were thinking during their dental cleaning, these self-talk strategies may have increased direction-following and cooperative behavior during treatment in our TD group. However, the ability to use this type of cognitive coping technique may be influenced by cognitive development [39]. In our study, the ASD group had significantly lower expressive communication scores than the TD group; as this scale is strongly correlated with IQ [40], our group of children with ASD may have not had sufficient cognitive/language abilities to utilize these types of verbally mediated cognitive coping strategies.

Uncooperative behavior in the TD group was correlated only with age, suggesting that the younger the child's age, the greater the uncooperative behavior. This supports previous research suggesting that younger children are less cooperative during dental treatment [8]. Based on past research, it was expected that dental anxiety would also be correlated with uncooperative behavior in these children, as high dental anxiety is often linked to uncooperative behavior in the literature [41]. However, with the constraints of the small sample size and only four TD children meeting the criteria for borderline (*n* = 3) and clinical (*n* = 1) dental anxiety based on the cut-off scores of Berge et al. [26], we did not have enough power or variability in dental anxiety to determine whether this relationship existed in our group.

In the ASD group, expressive communication was correlated with uncooperative behavior on both dentist- and researcher-report measures; this finding is consistent with research by Marshall et al. [3] which suggested that both receptive and expressive language difficulties were associated with uncooperative behavior. However, contrary to past findings in children with ASD [3, 8], age was not correlated with uncooperative behavior, although age did approach significance in its correlation with physiological distress.

These findings highlight that new strategies may be required to decrease uncooperative behavior in children with ASD in the dental clinic, compared to those recommended for typically developing children. For instance, since impairments in communication are highly prevalent in ASD [6] and lower expressive communication ability is correlated with uncooperative behavior, steps need to be taken to overcome this obstacle. This barrier is twofold: the dentist's ability to give directions that the child understands may be limited, and the child's ability to communicate choices, needs, fears, and pain to the dentist may be likewise impaired. In addition, even verbally fluent children with ASD have impairments in nonverbal communication and the interpretation of nonverbal social cues [6]. Therefore, conventional behavioral management strategies that rely on communication such as tell-show-do, voice control, nonverbal communication, and verbal positive reinforcement [42] may not be as successful with children with ASD. Accommodations such as using picture schedules or boards, social stories to prepare children for dental visits, visual aids, speaking in short, concise phrases that are repeated often, behavioral training and modeling, and/or desensitization appointments may be helpful with this population. Some of these techniques have already been utilized for children with ASD during dental care and in other arenas with success [5, 14, 43–46]. Additionally, adapting the dental environment to decrease arousal may be beneficial for the ASD population, as physiological and behavioral distress were found to be moderately to strongly correlated in this study.

For instance, Shapiro et al. [36] adapted the visual, auditory, somatosensory, and tactile stimuli of the dental environment to decrease arousal and uncooperative behaviors in children with developmental disabilities (not ASD). Other suggestions to diminish the aversive nature of the stimuli experienced in the dental office include adaptations to sensory stimuli encountered, including visual stimuli (wearing sunglasses, dim lights, and avoiding light shining in eyes), auditory stimuli (listen to music on headphones and wear earmuffs or an ear-covering hat), gustatory stimuli (allow more frequent rinsing of paste and use no-taste products such as pumice), and vestibular/movement stimuli (have child climb into an already fully reclined dental chair) [47]. Lastly, tactile “deep pressure” stimuli which produce a calming effect [34] could be helpful in the dental office; one could lay a weighted blanket or even a traditional X-ray vest over the child's chest to provide this deep pressure [36, 47].

There are several limitations to this study. Due to our small sample size group differences and/or variable correlations may have been masked. Children with ASD are a heterogeneous group; therefore, in this pilot study of 22 participants with ASD some differences in the population may not be evident. The majority of the children with ASD in this study were low functioning in terms of their level of expressive language, so findings may not generalize to children with ASD with more verbal skill. The physiological data of the children in both groups also varied largely, making it difficult for statistical tests to capture a difference. Additionally, due to the non-experimental, correlational nature of this study, causality of the relationship between physiological and overt behavioral distress and child factors cannot be determined. Lastly, our study was conducted at a dental clinic in a teaching hospital whose mission includes serving children with disabilities; therefore, the dental providers may have had more skill than providers in the general community and thus minimized our results. Despite these limitations, this study adds to the understanding of the oral care experiences of children with ASD, specifically as they relate to behavioral and physiological distress and their potential correlates. As the prevalence of ASD is significantly higher today than in the past, estimated in 2014 to be approximately 1 in 68 children in the U.S. [35], more dentists will encounter children with ASD in their practice. It is therefore of the utmost importance to be aware of the differing experiences of children with ASD during oral care.

## 5. Conclusions

Based on this study's results, the following conclusions can be made.

1. Children with ASD exhibit significantly more uncooperative behaviors during routine dental cleanings compared to typically developing children.
2. Children with ASD exhibit significantly higher electrodermal arousal (non-specific skin conductance response frequency) compared to TD children, indicating greater physiological stress during dental cleaning.
3. Physiological stress (as measured by non-specific skin conductance response frequency) is significantly correlated with overt behavioral distress in children with ASD, indicating that as physiological stress increases so does behavioral distress.
4. Younger age is correlated with uncooperative behavior in typically developing children; in children with ASD, lower expressive communication ability and physiological distress are correlated with uncooperative behavior.

## Figures and Tables

**Table 1:** Descriptive characteristics of TD and ASD groups.

Descriptive characteristics	TD (*n* = 22)	ASD (*n* = 22)
	Mean (SD)	Mean (SD)
Age	8.3 (2.1)	8.2 (1.9)
Short sensory profile∗∗	164.3 (12.9)	114.2 (22.0)
Child and Adolescent Symptom Inventory-Anxiety Scale∗∗	7.2 (5.90)	18.6 (9.10)

	*n* (%)	*n* (%)

Gender∗		
Male	10 (45.5)	18 (81.8)
Female	12 (54.5)	4 (18.2)
Race		
Caucasian	18 (81.8)	21 (95.5)
Not Caucasian	4 (18.2)	1 (4.5)
Ethnicity		
Not Hispanic, not Latino	7 (31.8)	4 (18.2)
Hispanic, Latino	15 (68.2)	18 (81.8)
Maternal education level		
High school, GED, or less	9 (40.9)	4 (18.2)
Vocational/associates/college courses	6 (27.3)	15 (68.2)
Bachelor's degree or more	7 (31.8)	3 (13.6)
Paternal education level^a^		
High school, GED, or less	7 (33.3)	11 (52.4)
Vocational/associates/college courses	7 (33.3)	5 (23.8)
Bachelor's degree or more	7 (33.3)	5 (23.8)
VABS-II expressive language subtest of communication domain (adaptive level)∗∗		
Low (≤2 percentile rank)	0 (0.0)	9 (40.9)
Moderately low (3–17 percentile rank)	3 (13.6)	11 (50.0)
Adequate (18–83 percentile rank)	14 (63.6)	2 (9.1)
Moderately high (84–94 percentile rank)	5 (22.7)	0 (0.0)
High (≥98 percentile rank)	0 (0.0)	0 (0.0)
Children's Fear Survey Schedule-Dental Subscale (total score)∗∗		
Nonclinical range	18 (81.8)	6 (27.3)
Borderline range	3 (13.6)	5 (22.7)
Clinical range	1 (4.5)	11 (50.0)

^a^Missing data (*n* = 1 ASD group; *n* = 1 TD group); mother did not answer question.

*P≤ .05, **P ≤ .001.

**Table 2:** Differences in behavior and physiological distress variables.

Distress variable	TD Mean ± SD	ASD Mean ± SD	Effect size^a^
Anxiety and Cooperation Scale (continuous)	0.45 ± 1.06	2.07 ± 1.59	1.3∗∗
Children's Dental Behavior Rating Scale (scale score)	34.69 (12.47)	47.31 (8.61)	1.1∗∗
Skin conductance level^b^			
Baseline	4.39 ± 3.51	4.55 ± 2.87	0.1
Oral examination	4.14 ± 3.65	5.44 ± 3.77	0.3
Prophylaxis	4.35 ± 4.15	5.66 ± 3.96	0.4
Fluoride application	3.97 ± 4.22	6.24 ± 4.23	0.7∗
Total dental cleaning (exam, prophylaxis, and fluoride)	4.22 ± 3.87	5.63 ± 3.85	0.40
Frequency of non-specific skin conductance responses			
Baseline	5.60 ± 3.99	6.68 ± 3.64	0.4
Oral examination	3.03 ± 2.13	6.90 ± 4.48	1.1∗∗
Prophylaxis	3.40 ± 3.10	5.90 ± 3.40	0.7∗
Fluoride application	1.77 ± 2.76	4.80 ± 4.21	0.6∗
Total dental cleaning (exam, prophylaxis, and fluoride)	3.0 ± 3.3	5.8 ± 3.3	1.0∗∗

	*n* (%)	*n* (%)	Odds Ratio^a^

Frankl Scale			11.2∗
Negative behavior (1-2)	2 (9%)	10 (46%)	
Positive behavior (3-4)	20 (91%)	12 (54%)	
Presence of restraining hands	2 (9.1%)	16 (72.7%)	28.5∗∗

^a^Effect size is the effect of the group on the variable, adjusted for order of visit. Cohen's *d* used for continuous variables and odds ratio for dichotomous variables.

^b^While raw scores are presented here, analyses were performed on log values.

*P ≤ .05, **P ≤ .001.

**Table 3:** Correlations between behavioral and physiological measures of distress and descriptive variables in TD and ASD groups.

	A & C Scale	Frankl Scale	CDBRS	Hands	CFSS-DS	CASI-Anx	VABS-II	SSP	Age	SCL	NS-SCR
A & C Scale		−0.96∗∗∗	0.84∗∗∗	0.81∗∗∗	0.27	0.12	−0.42∗∗	−0.32	−0.003	0.04	0.62∗∗
Frankl Scale	−0.91∗∗∗		−0.84∗∗∗	−0.83∗∗∗	−0.18	−0.18	0.44∗∗	0.27	0.02	0.11	−0.57∗∗
CDBRS	0.63∗∗	−0.66∗∗∗		0.82∗∗∗	0.28	0.30	−0.34^†^	−0.35^†^	−0.03	−0.09	0.43∗∗
Hands	0.68∗∗∗	−0.64∗∗∗	0.51∗∗		0.28	0.14	−0.68∗∗∗	−0.15	0.08	−0.11	−0.37∗
CFSS-DS	0.11	−0.25	0.26	−0.004		0.41∗	0.42	−0.43∗∗	0.07	−0.06	0.11
CASI-Anx	0.04	0.03	0.03	−0.14	0.36∗		−0.04	−0.61∗∗	0.11	0.18	0.11
VABS-II	−0.08	−0.03	−0.10	−0.04	−0.11	−0.58∗∗		0.01	−0.18	0.12	−0.07
SSP	−0.23	0.17	−0.20	−0.04	0.56∗∗	−0.75∗∗∗	0.57∗∗		0.40∗	−0.13	−0.29
Age	−0.38∗	0.42∗∗	−0.13	−0.27	−0.48∗∗	−0.24	0.17	−0.14		−0.31	−0.35^†^
SCL	−0.37∗	0.22	−0.11	−0.21	0.38∗	0.005	0.06	0.18	0.04		0.48∗∗
NS-SCR	−0.22	0.05	−0.10	0.05	0.32	0.36∗	−0.18	−0.14	−0.05	0.52∗∗	

Note. The lower left half of the matrix depicts the findings for the TD group and the upper right half represents the ASD group. A & C Scale: Anxiety and Cooperation Scale (behavioral distress); CDBRS: Children's Dental Behavior Rating Scale (behavioral distress); Hands: use of restraining hands during dental cleaning (behavioral distress); CFSS-DS: Children's Fear Survey Schedule-Dental Subscale (dental anxiety); CASI-Anx: Child and Adolescent Symptom Inventory-Anxiety Scale (general anxiety); VABS-II: Expressive Language Subtest of the Communication Domain of the Vineland Adaptive Behavior Scales II (communication ability); SSP: Short Sensory Profile (sensory processing difficulty); SCL: skin conductance level throughout dental cleaning (physiological distress); NS-SCR: frequency of non-specific skin conductance responses throughout dental cleaning (physiological distress).

*P ≤ .10, **P ≤ .05, ***P ≤ .001, ^†^approaching significance.
